# Treatment of Bone Defects and Nonunion via Novel Delivery Mechanisms, Growth Factors, and Stem Cells: A Review

**DOI:** 10.1021/acsbiomaterials.4c01279

**Published:** 2024-11-11

**Authors:** Quinn T. Ehlen, Joseph P. Costello, Nicholas A. Mirsky, Blaire V. Slavin, Marcelo Parra, Albert Ptashnik, Vasudev Vivekanand Nayak, Paulo G. Coelho, Lukasz Witek

**Affiliations:** ∇ University of Miami Miller School of Medicine, Miami, Florida 33136, United States; ‡ Center of Excellence in Morphological and Surgical Studies (CEMyQ), Faculty of Medicine, Universidad de La Frontera, Temuco 4811230, Chile; § Department of Biochemistry and Molecular Biology, University of Miami Miller School of Medicine, Miami, Florida 33136, United States; ∥ Biomaterials Division, NYU Dentistry, New York, New York 10010, United States; ⊥ Division of Plastic Surgery, DeWitt Daughtry Family Department of Surgery, University of Miami Miller School of Medicine, Miami, Florida 33136, United States; # Department of Biomedical Engineering, NYU Tandon School of Engineering, Brooklyn, New York 11201, United States; 7 Hansjörg Wyss Department of Plastic Surgery, NYU Grossman School of Medicine, New York, New York 10016, United States; □ Department of Comprehensive Adult Dentistry, Faculty of Dentistry, Universidad de La Frontera, Temuco 4811230, Chile

**Keywords:** Fracture nonunion, growth factors, stem cells, bone regeneration

## Abstract

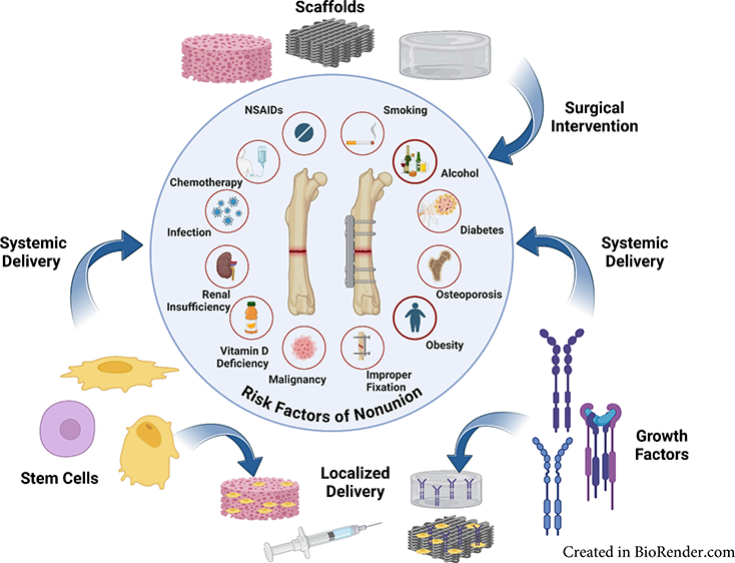

Bone nonunion following a fracture represents a significant global healthcare challenge, with an overall incidence ranging between 2 and 10% of all fractures. The management of nonunion is not only financially prohibitive but often necessitates invasive surgical interventions. This comprehensive manuscript aims to provide an extensive review of the published literature involving growth factors, stem cells, and novel delivery mechanisms for the treatment of fracture nonunion. Key growth factors involved in bone healing have been extensively studied, including bone morphogenic protein (BMP), vascular endothelial growth factor (VEGF), and platelet-derived growth factor. This review includes both preclinical and clinical studies that evaluated the role of growth factors in acute and chronic nonunion. Overall, these studies revealed promising bridging and fracture union rates but also elucidated complications such as heterotopic ossification and inferior mechanical properties associated with chronic nonunion. Stem cells, particularly mesenchymal stem cells (MSCs), are an extensively studied topic in the treatment of nonunion. A literature search identified articles that demonstrated improved healing responses, osteogenic capacity, and vascularization of fractures due to the presence of MSCs. Furthermore, this review addresses novel mechanisms and materials being researched to deliver these growth factors and stem cells to nonunion sites, including natural/synthetic polymers and bioceramics. The specific mechanisms explored in this review include BMP-induced osteoblast differentiation, VEGF-mediated angiogenesis, and the role of MSCs in multilineage differentiation and paracrine signaling. While these therapeutic modalities exhibit substantial preclinical promise in treating fracture nonunion, there remains a need for further research, particularly in chronic nonunion and large animal models. This paper seeks to identify such translational hurdles which must be addressed in order to progress the aforementioned treatments from the lab to the clinical setting.

## 1 Introduction

Nonunion, or the body’s inability to heal a fracture, is a known complication following acute fractures.^1^ Although a consensus for nonunion has been lacking, according to the United States Food and Drug Administration (FDA), a nonunion is defined by the presence of an unhealed fracture for a minimum of nine months, with no healing progression for three months.^2,3^ While many acute fractures result in adequate healing with proper realignment of the bone cortices, approximately 2–10% of all fractures have been indicated to result in nonunion.^4,5^ The transition from fracture to nonunion involves several stages of healing that can be disrupted. Initially, a hematoma forms at the fracture site, followed by inflammation and recruitment of cells necessary for healing. During the repair phase, a soft callus forms, which later ossifies into a hard callus. Nonunion can occur when there is insufficient blood supply, excessive motion at the fracture site, or chronic inflammation, preventing proper callus formation and healing.^6^ While nonunion account for only a small subset of all fractures, its treatment represents a significant financial burden with direct costs often exceeding $25,000 per case.^7^ A nonunion can occur at any anatomical location, with the tibia and fibula presenting with the highest rates (2.0–9.2% and 0.3–5.4%, respectively).^8^ Furthermore, with age, the femur and pelvis are also likely to become the most common sites of nonunion.^9^

**Figure 1:**
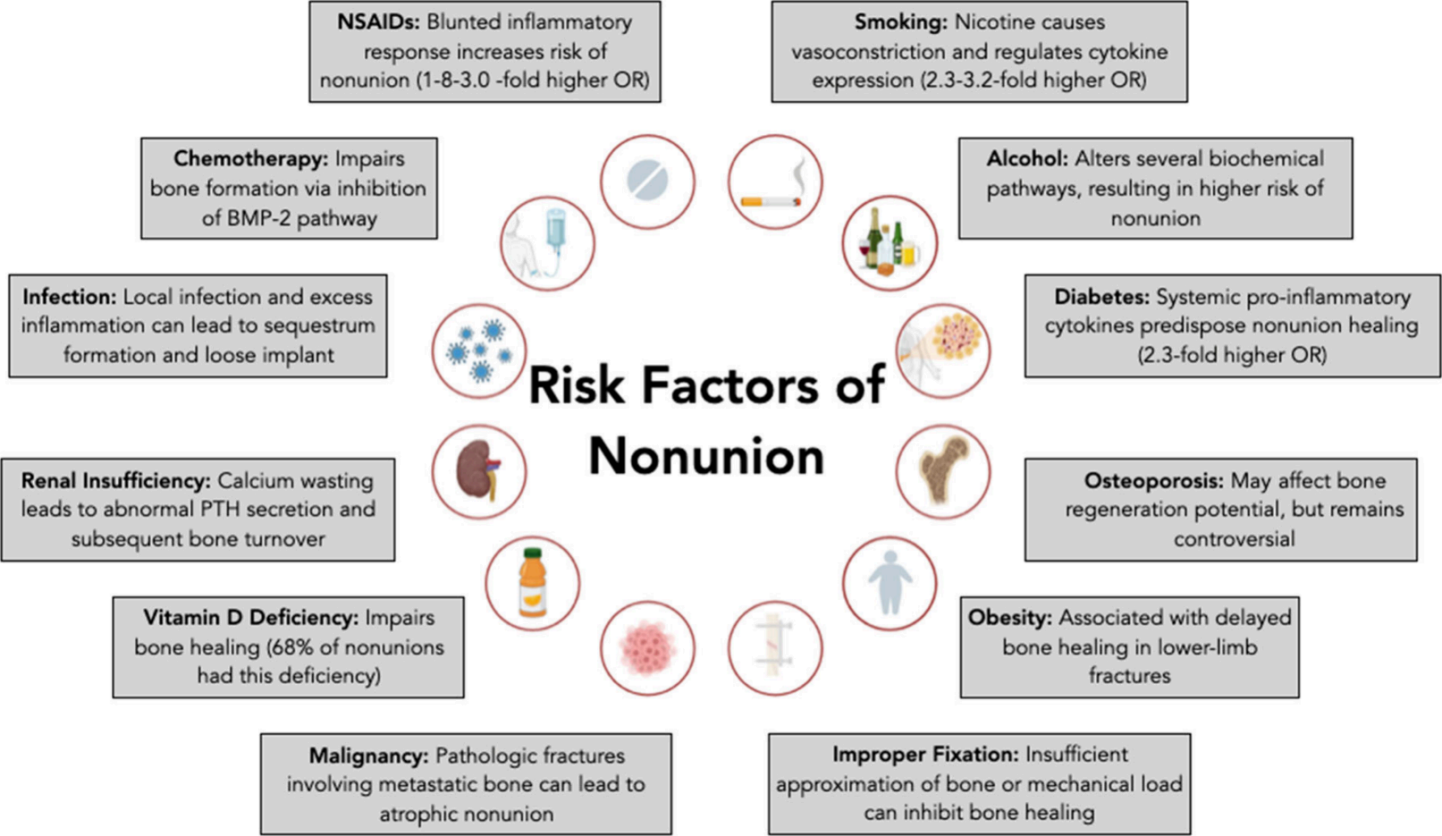
Risk factors that can contribute to nonunion during bone healing. Schematic generated on Biorender.com.

In addition to age, there are several other risk factors for nonunion events, such as but not limited to traumatic mechanisms of injury, smoking, nonsteroidal anti-inflammatory drug (NSAID) use, infection, and failed internal fixation (Figure 1).^10^ Further risk factors include bones with a naturally tenuous blood supply such as the scaphoid, hamate, and talar neck.^9^ The incidence of nonunion is therefore multifactorial, as the environment for proper bone healing is influenced by both biological (i.e., adequate blood supply, medications, past medical history) and mechanical (i.e., fracture approximation, physical stress to the fracture site) components.^9,11^ Although numerous risk factors have been identified, medical therapies aimed to prevent nonunion have been limited due to the complex nature of fracture healing.

Fracture healing occurs through two distinct processes: intramembranous and endochondral ossification. Intramembranous ossification, primarily observed in flat bones or during stable fracture healing, involves direct bone formation by osteoblasts.^12^ In contrast, endochondral ossification, primarily observed in long bones and unstable fractures, proceeds through a cartilage intermediate prior to bone formation.^12^ Understanding these processes is crucial for treatment planning, as the type of ossification has been reported to influence the choice of growth factors, scaffolds, and delivery mechanisms.^13^ For instance, biomaterials designed for long bone regeneration may need to support both cartilage formation and subsequent ossification, while those for flat bones can be tailored to enable direct osteoblast activation.^13^

Furthermore, reactive oxygen species (ROS) and oxidative stress play a critical role in bone healing and can significantly impact the progression of fracture repair. While controlled levels of ROS are necessary for normal cellular function and signaling during the healing process, excessive ROS production can lead to oxidative stress, which impairs bone formation and delays union.^14^ Oxidative stress can damage cellular components, including proteins, lipids, and DNA, leading to cellular dysfunction and apoptosis of osteoblasts and osteocytes.^15^ Furthermore, increased oxidative stress can increase osteoclast differentiation and activity, promoting bone resorption.^14^ In the context of nonunion, persistent oxidative stress can create a chemical barrier that hinders the progression of bone formation and contributes to delayed healing. Understanding and addressing the role of ROS and oxidative stress in nonunion is therefore also crucial for developing effective treatment strategies and may provide new targets for therapeutic interventions.

**Figure 2:**
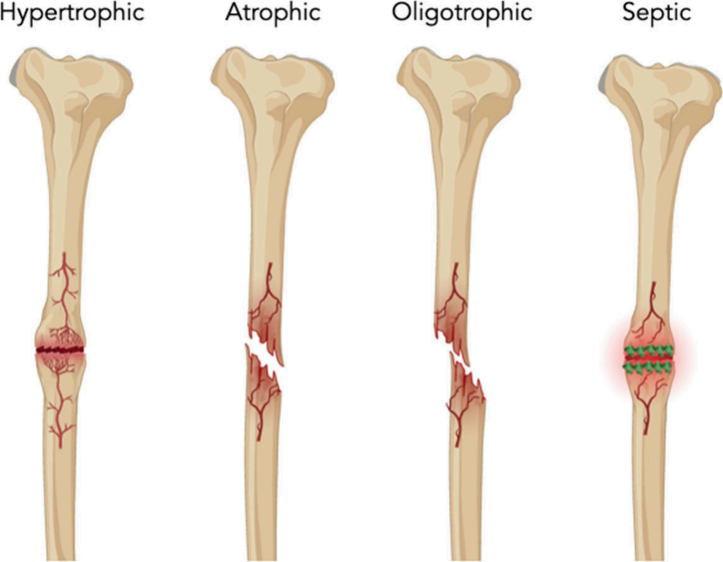
Schematic depicting the common types of nonunion. Schematic generated on Biorender.com.

Conservative management of nonunion have been indicated with nonoperative treatment such as pulsatile ultrasound stimulation or fracture braces, yet rarely adequate.^1,16^ Therefore, the majority of cases pertaining to nonunion treatment require surgical intervention. The appropriate operative procedure is contingent upon the type of nonunion present. Broadly, there are four categories, (i) hypertrophic, (ii) atrophic, (iii) oligotrophic, and (iv) septic nonunion (Figure 2).^1,17^ Hypertrophic nonunion refers to abundant callus with no bone bridging, insinuating adequate blood supply with inadequate mechanical stability.^18^ Atrophic nonunion refers to absent callus alluding to inadequate blood supply or improper fixation.^18^ Oligotrophic nonunion lies in between hypertrophic and atrophic and suggests inadequate reduction.^18^ Finally, septic nonunion is caused by an insufficient nutritional environment due to microorganism consumption (Figure 2).^18^ Regardless of type, once a surgical treatment plan is selected there still remains a debate over ideal timing for intervention.^19^

In conjunction with surgery, biologics—primarily growth factors and stem cells, are often supplemented to facilitate bone healing and have become critical components in the treatment of nonunion.^20^ The purpose of biologic use, postoperatively, is to increase osteogenesis, osteoinduction, and osteoconduction to promote the regeneration of new, healthy bone. The application of biologics to an area of a nonunion in clinical practice generally occurs after several months of nonhealing at a fracture site.^20^ Historically, much of the research on biologics for the treatment of bone defects and nonunion has been performed using acute critically sized defects in animal models.^21−39^ By definition, a critically sized defect is one which healing will not occur spontaneously and will require surgical intervention.^40^ In preclinical, animal models, these defects are surgically induced and then treated with the biologic. However, such models do not allow adequate intermediate healing time without the use of the biologic (which is common of a human nonunion to form surrounding the site of the defect), possibly resulting in different efficacies for treatment.^41,42^ This lack of intermediate healing time results in models, which closer resemble large acute fractures instead of chronic nonunions. This translational hurdle must be addressed to progress toward clinical applications. As an alternative, to simulate bone nonunion, models should present an acute critically sized defect induced without immediate treatment. Following a designated healing period, an investigative biologic should be administered, consistent with the time course of clinical nonunion treatment.

The current review will focus on the treatment of long bone nonunion with growth factors and stem cells, as well as novel mechanisms of biologic delivery, while considering the acute versus chronic nonunion models in both animals and humans. Acute models consist of the traditional preclinical animal models in which a defect is created, and biologic treatment is administered simultaneously. Chronic nonunion models consist of a defect creation with allotted healing time, followed by treatment administration at a later time. The latter model more accurately represents the clinical timeline of a chronic nonunion. While previous review articles have focused on the role of a specific growth factor such as bone morphogenic protein (BMP), or the cellular environments of nonunion; to our knowledge, none have provided a comprehensive summary of these treatment modalities in both clinical and preclinical long bone models while also delineating the key difference of acute versus chronic settings.^3,43^

## 2 Novel Physical Delivery Mechanisms

While growth factor and stem cell research are critical areas of exploration in the treatment of nonunion, the techniques by which these therapies are effectively delivered to the site are equally as crucial. As an alternative to the conventional autogenous graft, with its respective drawbacks such as donor site morbidity, finite supply, prolonged operative times, and increased pain, researchers continue to investigate nonautologous alternatives to facilitate bone growth in the surgical treatment of nonunion. Scaffolds derived from bioceramics, natural or synthetic polymers, or a combination have been evaluated as delivery vehicles for growth factors and stem cells known to facilitate the regenerative process.^44^ The efficacy of each delivery mechanism depends upon the physical and chemical properties of the given biomaterial and their interaction with the surrounding environment *in vivo*. Depending upon scaffold geometry and composition, they have the potential to provide stability to the site of nonunion as well as promote osteoconduction, osseointegration, and angiogenesis. Recently, scaffolds have been developed utilizing computer aided design (CAD) and fabricated using 3D printing to create site-specific implants customizable, in certain cases, down to the nanoscale. Numerous studies (Table 1) have comprehensively evaluated the *in vitro* or *in vivo* performance of an array of scaffolds in pursuit of an ideal therapeutic delivery system. This section aims to discuss the benefits and drawbacks of these various mechanisms of delivery.

**Table 1:** Novel Mechanisms of Delivery and Materials for the Treatment of Nonunion in Long Bones

Author	Subjects	Age	Size of Defect	Product	Administration Time	Site of Nonunion	Results	References
Brown et al. (2011)	Rats (*n* = 100)	NA	6 mm	Biodegradable polyurethane (PUR) and PUR/microsphere composite scaffolds with varying BMP-2 release kinetics compared to collagen sponge delivery system.	Acute	Femur	The most efficacious technique was a PUR scaffold with a burst followed by a sustained release of BMP-2, which showed faster healing at 4 weeks.	(21)
Cho et al. (2017)	Humans (*n* = 21)	Avg. = 50.2 years	Avg. = 8.9 cm	Circumferential bone graft around absorbable gelatin sponge cores	Chronic	Tibia (*n* = 11), Femur (*n* = 8), Humerus (*n* = 2)	Successful healing in 18 out of 21 cases. For cases with limited availability of autogenous grafts, a circumferential bone graft around a sponge core is advantageous and effective.	(45)
Deininger et al. (2021)	Rats (*n* = 24)	12 weeks	5 mm	Silk scaffolds with an apatitic calcium phosphate coating and loaded with BMP-2	Acute	Femur	The silk-fibroin scaffold allowed for fracture healing with lower BMP-2 dosage.	(22)
Eriksson et al. (2021)	Rabbits (*n* = 18)	10–11 months	1 cm	Amorphous S53P4 bioactive glass (BAG) scaffolds	Acute	Femur	The BAG scaffold induced osteostimulative membranes and achieved stable scaffold integration.	(23)
Godbout et al. (2023)	Rats (*n* = 35)	NA	5 mm	Gelatin scaffold with endothelial progenitor cells (EPCs) delivered via culture medium (CM), phosphate-buffered saline, platelet-poor plasma (PPP), or platelet-rich plasma (PRP)	Acute	Femur	CM, phosphate-buffered saline, PPP, and PRP were all viable options to deliver EPCs to nonunion fractures, but phosphate-buffered saline is the cheapest and safest in humans.	(24)
Grgurevic et al. (2019)	Rabbits (*n* = 20)	10 weeks	17 mm	Autologous bone graft substitute containing BMP-6 within autologous blood coagulum (ABC) scaffold	Acute	Ulna	ABC scaffold with BMP-6 provided robust bone formation and complete bridging at a lower dose of BMP-6 compared to other scaffolds.	(25)
Hall et al. (2023)	Mice	NA	4 mm	Periosteum-derived cartilaginous spheroids combined with PCL meshes to create a biohybrid sheet	Acute	Tibia	Biohybrid implants were wrapped around the defects, which promoted healing with enhanced bioactivity.	(26)
Kaipel et al. (2014)	Rats (*n* = 27)	20–24 weeks	3.8 mm	Fibrin clots containing BMP2/7 plasmids with and without cationic polymer	Chronic (4 weeks)	Femur	Treatment with the BMP2/7 plasmids and the cationic polymer showed decreased bone volume. Cationic polymers are not suitable as promoters of BMP2/7 gene transfer.	(46)
Lauer et al. (2020)	Rats (*n* = 36)	10 weeks	6 mm	Porous polylactic acid (PLA) cylinder with collagen type I and either stromal-derived factor 1 (SDF-1) or BMP7	Acute	Femur	The 3D printed acellular biomaterial made of PLA with collagen type 1 and modified with SDF-1 had the best outcomes of bone regeneration.	(27)
Li et al. (2021)	Rabbits (*n* = 15)	NA	NA	β-TCP/polylactic-*co*-glycolic acid (CTP) scaffold with Carfilzomib (CFZ)	Acute	Radius	CFZ-loaded scaffolds can treat nonunion through osteogenesis and angiogenesis while inhibiting osteoclasts.	(28)
Murahashi et al. (2019)	Mice (*n* = 60)	8 weeks	3 mm	Poly(l-lactic acid) (PLLA) nanosheets loaded with fibroblast growth factor-2 (FGF-2)	Acute	Femur	The PLLA nanosheets displayed long-lasting, continuous release of FGF-2, enhancing bone regeneration.	(29)
Oest et al. (2007)	Rats (*n* = 28)	13 weeks	8 mm	Poly(l-lactide-*co*-d,l-lactide) (PLDL) scaffolds combined with RGD-alginate hydrogel with or without growth factors	Acute	Femur	PLDL scaffolds with both growth factors resulted in improved healing. However, the scaffold was slow-degrading, which may have limited bone integration.	(30)
Ozturk et al. (2013)	Rabbits (*n* = 30)	NA	10 mm	Hydroxyapatite-containing gelatin (cryogel) scaffold with and without local VEGF administration	Acute	Tibia	The cryogel scaffold yielded promising results, both with and without the application of VEGF, demonstrating better scores in all criteria compared to the control.	(31)
Pek et al. (2008)	Rats (Femur: *n* = 50) Pigs (Tibia: *n* = 10)	NA	5 mm, 2 cm	Porous bioresorbable nanocomposite bone scaffold made of collagen fibers and synthetic apatite nanocrystals	Acute	Femur (Rats), Tibia (Pigs)	The nanocomposite scaffold showed bioactivity and was osteoconductive to heal nonunion fractures.	(32)
Priddy et al. (2014)	Rats (*n* = 8)	13 weeks	8 mm	Nanofiber mesh with oxidized alginate hydrogel compared to irradiated alginate	Acute	Femur	The oxidized alginate hydrogel implant showed more diffused and fragmented healing relative to the irradiated alginate implant.	(33)
Schwarz et al. (2018)	Rats (*n* = 16)	12 weeks	5 mm	BMP-2 loaded on an absorbable sponge, but stabilized with 3 different external fixator stiffnesses	Acute	Femur	Flexible fixation showed prolonged chondrogenesis, but osteogenesis began earlier with rigid and semi-rigid fixation. Flexible fixation resulted in more bone formation at later time points.	(34)
Schwarz et al. (2012)	Rats (*n* = 32)	12 weeks	5 mm	Type I collagen scaffolds with BMP-2 with or without mechanical loading	Acute	Femur	Mechanical stimulation enhanced mineralized tissue and increased fibrous tissue. Both groups achieved bony bridging.	(35)
Veeresh et al. (2021)	Rabbits (*n* = 12)	12 weeks	1 cm	Chitosan/chondroitin sulfate/gelatin/nanobioglass scaffold	Acute	Ulna	The scaffold was efficient in osteoconduction and osteoinduction to heal the nonunion. It was also biocompatible and nonimmunogenic.	(36)
Viateau et al. (2006)	Sheep (*n* = 11)	2 years	25 mm	Morselized autologous corticocancellous graft	Chronic (6 weeks)	Metatarsal	This model resulted in complete nonunion healing, with bone that may have osteogenic properties	(47)
Wang et al. (2015)	Rats (*n* = 16)	NA	2.5 mm	PLLA core-poly(lactic-*co*-glycolic acid) (PLGA) shell double-walled microsphere loaded with FGF-2 and BMP-2, respectively	Acute	Tibia	The microsphere product provided a sequential delivery of FGF-2 and BMP-2, which accelerated the process of bone fracture healing.	(37)
Yu et al. (2020)	Rabbits (*n* = 18)	4 months	15 mm	Preosteoblast-derived matrix (pODM) adhered with BMSCs and wrapped around gelatin methacryloyl (GelMA) hydrogel	Acute	Radius	The pODM product integrated with GelMA, healed the nonunion with reconstruction of the medullary cavity.	(38)
Zaky et al. (2017)	Rabbits (*n* = 12)	NA	16 mm	Poly(glycerol sebacate) (PGS) scaffold	Acute	Ulna	PGS enhanced healing by recruiting host cells, transducing mechanical signals for differentiation, and semi-loading bone via its elasticity.	(39)

### 2.1 Natural Polymers

Natural polymers possess enhanced biocompatibility and biodegradability relative to their synthetic counterparts. Current materials under investigation include gelatin, collagen, silk, alginate, chitosan and hyaluronic acid, each with varying fabrication methods.^48^ The biocompatibility of these aforementioned materials can promote osteogenesis and cellular adhesion. Furthermore, biodegradability of natural polymers may lead to improved osteointegration ability of implants, leading to their utility in bone tissue engineering.^44,49^ Additionally, these materials may contain beneficial functional groups on their surface, contributing to successful cellular differentiation and adhesion.^44^ Despite these advantages, polymers have also been associated with increased inflammation and immune response at the recipient site without additional vascularization benefits.^50^ Moreover, some materials have been found to contain pathogenic substances and increased degradation rates.^44^ Due to these reasons, investigators have attempted to combine natural polymers with other biomaterials in implant design for the treatment of bone nonunion, with variable results.

#### 2.1.1 Gelatin

Gelatin is a biodegradable polymer that is often harvested from bovine skin to produce a scaffold that is biocompatible and nontoxic.^51^ When used independently, this material dissolves rapidly in an aqueous environment, providing minimal use in bone regenerative procedures in which prolonged drug release is ideal.^52^ However, it is often used in combination with other biomaterials to serve as a flexible biodegradable scaffold. Cho et al. explored the ability of a gelatin sponge core, a natural polymer, for a circumferential bone graft to potentially reduce the autologous graft volume required, utilizing the well-established induced membrane technique.^45^ This technique involved a two-stage procedure where a cement spacer was initially placed to induce a vascularized membrane, followed by removal of the spacer and grafting in a second surgery.^45^

Traditional methods of tightly packed autologous grafts in circumferential defects have resulted in lack of revascularization of the graft core due to the increased distance from the outer membrane, precluding this region of the graft from obtaining blood supply and subsequent consolidation.^53^ Cho et al. theorized that the central portion of the graft could be replaced with a resorbable gelatin sponge to avoid central necrosis of the graft, while also reducing the autologous graft volume required from the patient.^45^ The gelatin-based sponge used, prepared from a purified type A pork gelatin, was biocompatible and biodegradable, leading to complete absorption within 4–6 weeks post implantation.^45^ Other studies utilizing gelatin sponges in bone reconstruction have demonstrated its successful implementation as a scaffold to support osteoblast proliferation, differentiation, and integration into the interior of the sponge, owing to its porous structure. The residuals of the sponge were incorporated into the surrounding bone, without any significant foreign body reaction.^54^ Godbout et al. further demonstrated the benefits of gelatin scaffolds in the treatment of nonunion, by utilizing this scaffold as a delivery vehicle for endothelial progenitor cells (EPCs) combined with various delivery media in a rat model of critically sized femoral defects.^24^ Gelatin scaffolds were successfully absorbed in the surrounding tissue, allowing for therapeutic delivery of EPCs. While the scaffolds itself were not the focus of the study, their biocompatible and biodegradable properties, as well as their ability to incorporate various media, were essential in facilitating investigation of the treatments of interest.

#### 2.1.2 Collagen

Collagen is another frequently utilized component of implantable scaffolds due to its organic origin and favorable mechanical properties. Natural bone consists of 30–35% collagen, allowing the scaffold to have both low immunogenicity and high absorbability.^32^ Additionally, because collagen is a structural protein, cells are prone to adhere to the scaffold to aid in bone regeneration. It is often combined with other materials, such as inorganics like calcium phosphates (CaP), or demineralized bone. These combinations have been demonstrated to mimic the chemical composition and microstructure of natural bone.^55^ Collagen has been frequently used in preclinical studies as a scaffold material to bridge nonunion fractures. For example, Schwarz et al. used two different rat femur critically sized defect models with a collagen sponge as the scaffold.^34,56^ They found success in bone regeneration; however, this was attributed to the loading of the sponge with BMP-2 rather than the collagen material itself. Additionally, studies analyzing the mechanical healing environment demonstrated that the stiffness of fixation and mechanical loading of the fracture can also impact bone volume and quality.^34,56^ Similarly, other studies have found success with BMP-2-loaded collagen sponges in combination with alendronate, which induces osteoblast differentiation and inhibits osteoclasts.^57^

Collagen can also be combined with Hydroxyapatite (HA) to form a more rigid implant, as seen in a study by Pek et al.^32^ This model combined Type I Collagen with nanocrystalline apatite to form a nanocomposite, which was then implanted into critically sized defects in rat femurs. This scaffold was composed of a collagen to apatite ratio identical to trabecular bone, with a similar molecular structure and crystallite size. The success of this study in bone healing was attributed to the excellent bioactivity of the bone-mimicking implant. Overall, these studies promote the use of collagen to deliver biologics and serve as a scaffold for the treatment of nonunion.

#### 2.1.3 Hydrogels

Natural polymeric materials can be structurally modified into highly hydrated polymer networks known as hydrogels. Hydrogels have been widely used as a scaffold for bone and cartilage defects, typically derived from natural sources.^58^ In addition to the benefits inherent in polysaccharides and proteins, hydrogel modification can improve biocompatibility as a result of its ability to simulate native extracellular matrix properties.^59,60^ However, despite beneficial characteristics of hydrogels, they can only be used when combined with other bioactive scaffolds and/or growth factors due to their osteogenic capacity and relatively low mechanical strength.^44^ Gelatin methacryloyl (GelMA) is one such hydrogel scaffold, derived from gelatin, which is widely used in bone repair due to its excellent biocompatibility and tunable physicochemical characteristics.^59,61^ Yu et al. attempted to combine a GelMA scaffold with a preosteoblast-derived matrix (pODM) tested in a rabbit model of critically sized radial bone defects to determine its capability to enable bone regeneration and prevent nonunion.^38^ In this induced membrane technique, GelMA was selected as the scaffold material due to its ability to degrade (versus a nonbiodegradable poly(methyl methacrylate) (PMMA) scaffold) and provide temporary mechanical support (versus a nonhydrogel, gelatin sponge).^38^

#### 2.1.4 Alginate

Alginate hydrogel scaffolds are natural polysaccharides derived from brown algae and bacteria. They are unique in their ability to be physically cross-linked at room temperature, enabling modification through a variety of techniques.^62^ Alginate is widely used in bone regeneration due to its biocompatibility and low cost. However, alginate scaffolds do not degrade readily in mammals, and therefore, must be modified via irradiation and/or oxidation to enable proper healing of the bony defect.^63^ Multiple studies previously examined irradiated alginate scaffolds for delivery of BMP-2 in critically sized rat bone defect models, facilitating enhanced bone regeneration over collagen sponges.^64^ However, portions of the scaffold were present after 12-weeks of healing, potentially impeding bone tissue formation and remodeling. Priddy et al. combined oxidation and irradiation of the alginate scaffold, to accelerate degradation while maintaining its appropriate functionality, thereby augmenting bone tissue formation and maturation in a similar critically sized rat bone defect model.^33^ According to the authors, this was the first study demonstrating prolonged bioactivity of BMP-2 retained within modified alginate hydrogels.^33^

#### 2.1.5 Silk

Silk fibroin is another natural polymer with advantageous characteristics including high biocompatibility, controlled biodegradability, tunable mechanical properties and osteointegrative ability.^65^ The versatility of this material allows for creation of optimal porosity of the scaffold, potentially improving cell migration into the defect area.^66^ Deininger et al. developed a biomimetic silk fibroin scaffold which enhanced rhBMP-2-mediated regeneration of critically sized bone defects in a rat model.^22^ Scaffolds were developed with anisotropic channel-like pores extending throughout the scaffold, which was then coated with a bone-like apatite, resulting in significantly improved osteogenic capacity. The authors concluded that the scaffold developed in this study allowed for a reduction of rhBMP-2 dosage, representing a promising option for the treatment of nonunion.^22^

### 2.2 Bioceramics

Bioceramics have been extensively studied for the reconstruction of long bone nonunion and bone regeneration. Bioceramics include inorganic materials which are biocompatible with a crystal structure, such as hydroxyapatite (HA), tricalcium phosphate (TCP), and bioactive glass, all of which have high melting points, electrical and corrosion resistances.^67^ Their high melting points allows them to be subjected to sterilization cycles without the concern for deterioration of shape or changes in mechanical properties. Furthermore, their electrical and corrosive resistances decrease the potential for unnatural biological reactions. One of the rationales behind using bioceramics is their ability to emulate natural bone and promote a healing environment while delivering various growth factors or stem cells. These materials are naturally osteointegrative, osteoinductive, and osteoconductive, providing a solid foundation for the formation of bone. Standalone, or when combined with additional biologics, they have been proven to stimulate osteogenesis and angiogenesis required for bone regeneration. Lastly, the various production methods of bioceramics can be advantageous for material manipulation and enhanced integration. For example, bioceramics can be 3D printed to customized shapes and finishes, which have been demonstrated to improve their utility.

#### 2.2.1 Tricalcium Phosphate

Calcium phosphate-based bioceramic, Tricalcium Phosphate (TCP), is associated with high compressive strength, rendering it resistant to deformation.^68^ Its composition and microstructure provides a strong bone-implant interface, which aids in its resorption process.^68^ It is often combined with different materials, including other bioceramics such as HA, natural polymers such as gelatin and poly(lactic-*co*-glycolic acid) (PLGA), and heavy metals such as silver.^68−72^ Overall, TCP has exhibited promising preclinical efficacy in bone regeneration of critically sized defects. For example, Tovar et al. studied the efficacy of customized TCP implants in a rabbit radius model using robocasting, a 3D printing technique.^71^ It was determined that the customized implant stimulated progressive bone remodeling with mechanical properties homogeneous to native bone.^71^ Li et al. utilized a similar rabbit radius model to study the efficacy of a TCP/PLGA scaffolds which were treated with carfilzomib, a proteasome inhibitor.^28^ Carfilzomib has been shown to inhibit osteoclast activity and increase alkaline phosphatase activity.^73^ Its combination with a scaffold that emulates the natural bony environment demonstrated further osteoblast differentiation, osteoclast inhibition, and improved bone formation. It is theorized that the porous nature of the scaffold allowed for the sustained release of carfilzomib, prolonging its effect over the healing period. The ability for the TCP/PLGA scaffold to be 3D printed with customized pore sizes allowed for optimal drug elution and improved bone regeneration. Similar studies using bioceramics have been performed in mice, rats, pigs, sheep, and dogs.^74^ A recent *in vitro* study by Pereira et al. also shows promise in using a type I collagen-TCP scaffold, which can be custom-printed using a direct ink writing technique.^75^ This combination scaffold displayed superior cell proliferation compared to either material, exclusively.^75^ However, *in vivo* studies are necessary to determine the clinical potential of this combination TCP bioceramic.

#### 2.2.2 Hydroxyapatite

Another common bioceramic material, HA, is frequently combined with other materials for scaffold fabrication. HA is a biocompatible, nontoxic, resorbable, osteoinductive, and osteoconductive material.^76^ However, when used alone, this material is fragile, hence its frequent combination with other materials. Guda et al. wrapped HA in collagen and implanted the scaffolds in long-bone defects in rabbits.^77^ It was found that the scaffolds coated with collagen outperformed scaffolds composed of HA alone. The combination treatment resulted in higher bone volume with density, strength, and toughness equivalent to autologous bone. The success of the scaffold was attributed to the porosity of HA along with its ability to serve as a barrier for excessive connective tissue growth causing fibrosis. Ozturk et al. used a slightly different implant in an HA-fortified gelatin scaffold,^31^ loaded with vascular endothelial growth factor (VEGF) and implanted into rabbit tibiae defects. The treatment group showed significantly improved bone healing relative to control, however, there were no signs of complete bone healing. While the authors largely relate the efficacy of the study to the stimulative properties of VEGF, they also mention the swellability of the scaffold to have allowed for the implant to mold into the structure of the defect, providing a suitable healing environment.^31^ Conversely, combining HA with collagen in a foam-like nanocomposite can yield varying mechanical properties. Pek et al. studied this scaffold, which proved to be osteoconductive in healing nonunion fractures.^32^ Their manufacturing process produced a scaffold with molecular structure and crystallite size similar to trabecular bone. Additionally, the implant had a mixture of ultrafine pores alongside larger pores, which are suspected to play a role in protein adhesion, cell attachment, and vascularization.^32^ Overall, the main utility of HA is its ability to be combined with many other biomolecules and be manufactured in various ways to promote an adequate healing environment.

#### 2.2.3 Bioactive Glass

Bioactive glass (BAG) is another class of bioceramic materials studied for the treatment of nonunion. This material is formed from silicates and coupled to other physiologic minerals such as calcium or phosphorus.^78^ Similar to HA and TCP-based scaffolds, BAGs are biocompatible, osteoconductive, porous and possess a tailorable rate of degradation.^78,79^ Eriksson et al. investigated an amorphous S53P4 BAG scaffold in acute critically sized defects in rabbit femurs.^23^ S53P4 specifically has been shown to have antimicrobial, osteoconductive, and osteointegrative traits.^80^ This rabbit model showed that the BAG scaffold induced osteostimulative membranes and achieved stable scaffold integration.^23^ The study claimed that this BAG produced continuous osteointegration due to its mechanical properties and upregulation of various BMPs, which have potential to eliminate the need for a two-operation approach to nonunion healing. S53P4 has also been used clinically by Tanner et al. using the two-operation approach.^81^ A PMMA spacer was placed to induce vascularization for 6 weeks followed by spacer removal and BAG implantation in the second procedure.^81^ The goal of the study was to determine noninferiority to autologous bone graft, however, it was later determined that BAG in combination with autologous bone may be the best material for treating nonunion.^81,82^ Finally, Veeresh et al. examined the role of a combination treatment with BAG, chitosan, chondroitin sulfate, and gelatin as a scaffold for treating rabbit ulna nonunion.^36^ The composite scaffold was found to result in both osteoconduction and osteoinduction in the nonunion model.^36^ The success of the study was attributed to properties of the combination treatment. Specifically, it was theorized that the pore size and slow degradation rate of the BAG was instrumental in adequate bone regeneration.

While bioceramics have shown promise in the treatment of chronic nonunion, some limitations remain. It has been reported that bioceramics alone can be brittle relative to other scaffold materials.^78^ Additionally, it is uncertain whether the efficacious results can be attributed to the bioceramic material itself or the architecture of the implant, or perhaps the manufacturing flexibility that contributes to the regenerative success of these materials.^67^ Nonetheless, there have been extensive studies, both preclinical and clinical, that suggest that bioceramics can be an effective mechanism of delivery in treating chronic nonunion. Many of its shortcomings can be overcome by combining bioceramics with other biomaterials, such as synthetic polymers, which exhibit different mechanical and biological properties.

### 2.3 Synthetic Polymers

Biocompatible and biodegradable synthetic polymers, such polycaprolactone (PCL), polylactic acid (PLA), polylactide-*co*-glycolide (PLGA) and polyurethanes (PU) are actively under investigation in the treatment of nonunion. Generally, synthetic polymers are readily available, cost-effective, and may be easily fabricated into three-dimensional defect-specific scaffolds.^83,84^ For applications in nonunion, specifically, they have the potential to provide timely structural support during the critical period of bone regeneration and remodeling prior to their degradation.^85^ For these reasons, they serve as excellent vehicles for stem cells and growth factors to enhance osteoconductivity at the site of nonunion.^21^ Despite many commonalities, each synthetic polymer exhibits nuanced differences in their resorption kinetics, metabolic profiles, and mechanical properties. Ultimately, the aim is to identify a polymeric formulation capable of withstanding load-bearing forces during healing of the nonunion.

#### 2.3.1 Polycaprolactone

Polycaprolactone (PCL) is a thermoplastic polyester with great potential to serve as a scaffold for bone regeneration within critically sized defects of long bones. Given its hydrophobicity, PCL exhibits an estimated degradation rate between one to two years.^86^ While many researchers find this extended degradation profile to interfere with bone regeneration, Hall et al. suggested that it may be beneficial in the setting of nonunion due to a minimization of inflammation-inducing pH shifts which would otherwise interfere with bone healing.^26^ Hall et al. utilized a 3D-printed PCL scaffold to seed human periosteum derived cells, forming a regenerative biohybrid sheet. After culturing *in vitro* for 14 days, implantation of the biohybrid sheet within a critically sized murine tibia defect resulted in both mineralized bone and marrow formation capable of defect bridging.^26^ Notably, composite scaffolds comprised of PCL and ceramics (e.g., HA, CaP, calcium silicate) or natural polymers (e.g., collagen, gelatin, gliadin) enhance bioactivity, accelerate degradability, and improve mechanical strength.^87,88^ A study performed by Zhang et al. revealed that a 3D-printed magnesium calcium silicate/gliadin/polycaprolactone (MPGC) scaffold with high degrees of ceramic content exhibited a compressive strength higher than that of cancellous bone, in addition to improved osteogenesis compared to a scaffold with low ceramic content.^87^ PCL also has the capacity to exhibit shape memory effect.^89^ Shape memory polymers (SMPs) are unique such that upon activation from an external stimulus (e.g., heat), they transform from a deformed/compacted structure to the original structure or shape, allowing for minimally invasive surgical implantation.^86^

#### 2.3.2 Polylactic Acid, Polyglycolic Acid, and Polylactide-*co*-glycolide

Polylactic acid (PLA) and polyglycolic acid (PGA) also belong within the class of thermoplastic polyesters. Unlike PCL, PLA and PGA are hydrolyzed into acidic byproducts that may trigger a local inflammatory response.^86,90,91^ However, PLA and PGA continue to be applied in nonunion research as they are inexpensive and readily customizable.^27^ PLA is a hydrophobic polymer with an *in vivo* resorption time of 6–24 months.^30,92,93^ Whereas PGA, a hydrophilic polymer, presents with an accelerated degradation of 1.5–3 months *in vivo*.^92,93^ While PLA is often used alone in the setting of bone regeneration, PGA is rarely utilized independently as its expedited degradation provides inadequate mechanical support to a bone defect. In direct comparison, PLA offers slower degradation and higher mechanical strength, but its hydrophobic nature may limit cell attachment. In an attempt to combine the two material’s advantages, PLGA was developed, which is the copolymerized form of PLA and PGA, within which different ratios of PLA and PGA lead to drastic changes in degradability.^90,93^ With respect to PLA, it has been recently studied in critically sized mouse femoral defects in the form of multilayered nanosheets loaded with recombinant human fibroblast growth factor-2 (rhFGF-2).^29^ When fixed within the femoral defect by an intramedullary pin, the trilayered nanosheet group allowed for extended and controlled release of rhFGF-2 that was superior to the monolayered group. Ultimately, this resulted in a significant reduction in fracture gap and significant increase in bone volume between 4 and 8 weeks.^29^ Similarly, as detailed in an earlier section, PLGA was 3D-printed with β-TCP as a TCP/PLGA scaffold to enable extended release of carfilzomib (CFZ), a proteosome inhibitor, within nonunion rabbit radii defects.^28^ This study found that the sustained release of CFZ promoted enhanced osteogenesis and angiogenesis, while inhibiting osteoclastogenesis after 12 weeks *in vivo*.

#### 2.3.3 Polyurethanes

Polyurethanes (PU) are a family of elastomeric, thermoplastic polymers with properties widely suited for orthopedic applications.^94^ PU synthesis, from a polyester or polyether polyol and a chain extender, generates a segmented polymer with both hard (i.e., rigid) and soft (i.e., mobile) segments.^84^ The interchangeability of substrates in PU synthesis allows for its development as both an injectable and a scaffold with tunable *in vivo* degradation.^21,84,85,95^ The versatility of this class of synthetic polymers is unparalleled relative to its counterparts, such as PCL, PLA or PLGA.^96^ With respect to its role in the treatment of nonunion, porous PU scaffolds have been studied preclinically as a vehicle for controlled rhBMP-2 delivery. In comparison to the standard collagen sponge, the PU scaffold resulted in a burst, followed by sustained release of rhBMP-2 over a period of weeks, translating to significantly improved bone regeneration outcomes.^21^ In addition, an oxygen-releasing form of PU has been preclinically evaluated in combination with collagen as a periosteum-mimicking membrane for guided bone regeneration in the nonunion setting. To elaborate, when paired with a bone substitute, the PU-based membrane augmented bone and periosteal regeneration in a critically sized rat tibia model.^97^ PU has also been evaluated as an SMP. When placed within a load-bearing, critically sized rabbit femoral defect, *in vivo* shape-recovery activation of a PU-based scaffold was shown to generate a force that promoted bone ingrowth and neovascularization.^98^

### 2.4 Comparative Analysis of Delivery Mechanisms

While each delivery mechanism offers unique advantages, critical comparisons reveal important distinctions. Natural polymers like gelatin and collagen offer superior biodegradability and biocompatibility compared to synthetic polymers and bioceramics, but may lack mechanical strength for load-bearing applications.^48^ Bioceramics generally provide superior mechanical strength and can be engineered to release ions that promote osteogenesis. Hydrogels, while highly biocompatible and customizable, often require combination with other materials to achieve sufficient mechanical properties.^44^ Synthetic polymers and some bioceramics offer more controllable release profiles for growth factors.^21^ 3D-printed scaffolds, regardless of material, offer the highest degree of customization for patient-specific applications. Each delivery mechanism presents a trade-off between these factors, and the optimal choice depends on the specific requirements of the nonunion being treated. Future research should focus on combining the strengths of different materials to create hybrid delivery systems that optimize bone regeneration outcomes.

## 3 Growth Factors

**Figure 3:**
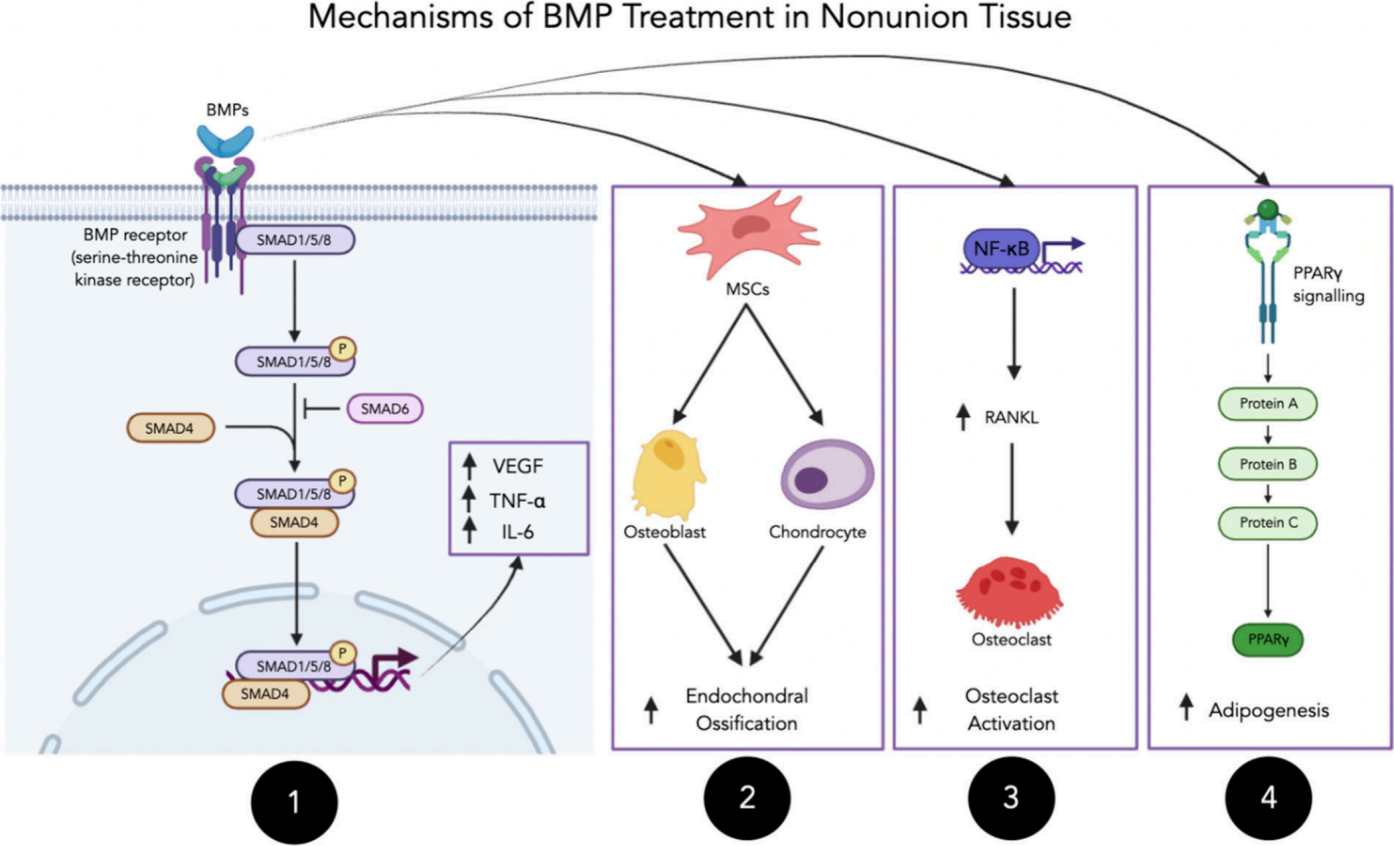
1) BMPs activates the SMAD pathway, which increases inflammatory cytokines VEGF, tumor necrosis factor alpha (TNF-α), and Interleukin-6 (IL-6).^104^ 2) BMPs recruit mesenchymal stem cells (MSCs), which are differentiated into osteoblasts and chondrocytes, leading to increased endochondral ossification.^105^ 3) BMPs induce the nuclear factor kappa-light-chain-enhancer of activated B cells (NF-κB) pathway, which increases receptor activator of nuclear factor-κB ligand (RANKL) and stimulates osteoclast activation.^106^ 4) BMPs induce the peroxisome proliferator-activated receptor gamma (PPARγ) signaling pathway, which increases adipogenesis and fat deposition.^107^ Schematic generated on Biorender.com.

Growth factors are biological molecules which have the capacity to influence their local environment and serve to stimulate the development of surrounding cells.^99^ Due to the importance of the local biologic environment in healing bone, growth factors have been postulated as one potential treatment for both large, acute bone defects as well as chronic nonunion. Growth factors can be subdivided into many different types for the purposes of classification.^100^ BMP-2 is one such potent osteoinductive growth factor that plays a crucial role in bone formation and repair. It belongs to the transforming growth factor-beta (TGF-β) family and is known for its ability to stimulate the differentiation of mesenchymal stem cells into osteoblasts. In the context of nonunion treatment, BMP-2 is particularly valuable due to its capacity to induce new bone formation, even in challenging environments where natural healing processes have failed. BMP-2 acts by binding to specific cell surface receptors, initiating a signaling cascade that ultimately leads to the expression of genes associated with osteoblast differentiation and bone matrix production (Figure 3).^101^ Though BMP has received majority of the attention, there has also been investigation into the use of other growth factors such as vascular endothelial growth factor (VEGF) and platelet derived growth factor (PDGF) (Table 2). VEGF is primarily known for its role in angiogenesis. In the context of nonunion treatment, VEGF is critical because adequate vascularization is essential for successful bone healing. Moreover, VEGF has been shown to have direct effects on osteoblasts and osteoclasts, further contributing to bone remodeling and regeneration.^102,103^

**Table 2:** Growth Factors for the Treatment of Nonunion in Long Bones

Author	Subjects	Age	Size of Defect	Experimental Groups	Administration Time	Site of Nonunion	Results	References
Bosemark et al. (2015)	Rats (*n* = 40)	10 weeks	6 mm	Tricalcium phosphate-hydroxyapatite (TCP-HA) scaffold	Chronic (4 weeks)	Femur	The combination treatment of TCP-HA scaffold + BMP-7 + systemic bisphosphonate resulted in the best healing outcomes for the critically sized defect.	(108)
				BMP-7				
				TCP-HA scaffold + BMP-7				
				TCP-HA scaffold + BMP-7 + systemic bisphosphonate				
Beck et al. (1998)	Rabbits (*n* = 14)	NA	1.5 cm	Autogenous cortical bone graft ± TGF-β with autologous bone marrow	Acute	Radius	Bone marrow with TGF-β produced healing of defects similar to autogenous cortical bone.	(109)
Bolander et al. (2017)	Mice (*n* = 13)	NA	4 mm	Human periosteum-derived cells in collagen type I gel with BMP-2	Acute	Tibia	BMP-2 treatment with periosteum-derived microtissues resulted in tissue formation that mimics natural fracture healing.	(110)
Cheng et al. (2019)	Rats (*n* = 34)	13 weeks	8 mm	PCL nanofiber mesh with human BMP-2 treatment	Acute | Chronic (8 weeks)	Femur	Delayed treatment resulted in decreased bone formation, reduced mechanical strength, and signs of chronic immune dysregulation.	(42)
Crawford et al. (2009)	Human (*n* = 9)	Variable	Variable	Locking plate and BMP	Chronic	Humerus	8 out of 9 patients showed signs of union at 1 year.	(111)
DeBaun et al. (2022)	Rats (*n* = 40)	8 weeks	8 mm	PCL/β-TCP scaffold surrounded by Collagen type I sheet with microspheres of BMP-2 or PDGF | Bare PCL/β-TCP scaffold	Acute | Chronic (4 weeks)	Femur	Complete unions were only formed with BMP-2 eluting membranes. Other treatment groups showed better healing than negative control.	(102)
Donegan et al. (2011)	Human (*n* = 11)	Variable	4 to 15 cm	Masquelet technique with varying combinations of BMP-2 and platelet rich concentrate	Acute *n* = 5Chronic *n* = 6	Femur = 5, Tibia = 6	10 of the 11 patients treated achieved radiographic consolidation and full weight bearing status.	(112)
Ekrol et al. (2008)	Human (recombinant human BMP (rhBMP)-7: *n* = 16, Autograft: *n* = 14)	Variable	Variable	Malunion corrective osteotomy: rhBMP-7 versus autogenous bone graft from ipsilateral iliac crest	Chronic (Avg. = 44 weeks)	Radius	rhBMP is less effective than autograft at healing osteotomies with internal or external fixation.	(113)
Franch et al. (2020)	Canine (*n* = 1)	1 year	NA	3D printed β-TCP scaffold with rhBMP-2 collagen sponges	Chronic (2 months)	Radius	Complete healing of the defect was observed 4 months after surgery and the bone plate was removed at 10 months.	(114)
Friedlaender et al. (2001)	Human (BMP-7+Col. I: *n* = 63, Autograft: *n* = 61)	BMP-7+Col. I: 38 years/Autograft: 34 years	NA	Internal fixation with BMP-7 and Collagen Type I (BMP-7+Col. I) versus internal fixation with autograft	Chronic (Median = BMP-7+Col. I: 27 months, Autograft: 33 months)	Tibia	The BMP-7 with Collagen type 1 had no donor site morbidity associated with autografts, yet had comparable rates of success to the autograft.	(115)
Freischmidt et al. (2021)	Rats (*n* = 34)	3 months	5 mm	Cerament G synthetic bone graft with gentamicin (HACaS+G) and administration of parathyroid hormone (PTH)	Chronic (5 weeks)	Femur	HACaS+G had osteoinductive and osteoconductive effects on the nonunion fracture. PTH was not osteoinductive, but increased vascularization.	(116)
Geiger et al. (2005)	Rabbits (*n* = 60)	6–9 months	15 mm	Collagen sponges with VEGF-plasmid transfected osteoblasts as a gene-activated matrix	Acute	Radius	The VEGF treatment significantly enhanced bone and vessel formation at the fracture site.	(103)
Giannoudis et al. (2009)	Human (*n* = 45)	Median = 43 years	NA	Autologous bone graft and rhBMP-7	Chronic (Median = 20 months)	Humerus (*n* = 7), Femur (*n* = 19), Tibia (*n* = 19)	Using autologous bone graft with rhBMP-7 may be more effective and economical than autologous bone graft alone.	(117)
Haffner-Luntzer et al. (2023)	Mice (*n* = 7)	12 weeks	1 mm	Recombinant Wnt1 in a collagen gel	Acute	Femur	The Wnt1 treatment had increased bone volume, and trabecular thickness compared to control group.	(118)
Heckman et al. (1999)	Canine (*n* = 30)	NA	3 mm	PLA and PGA copolymer with BMP, TGF-β, or both	Chronic (12–16 weeks)	Radius	The implants without BMP or TGF-β failed to heal the nonunion. The BMP treatment alone showed the largest increase in endosteal bone formation.	(119)
Hettiaratchi et al. (2020)	Rats (*n* = 41)	13 weeks	8 mm	PCL nanofiber mesh tube with BMP-2-loaded heparin microparticles	Acute	Femur	Heparin microparticles increased BMP-2 retention and improved spatial localization of bone formation.	(120)
Ihn et al. (2021)	Rats (*n* = 57)	12–14 weeks	6 mm	Matrix with various doses of BMP-2 transduced human bone marrow cells	Acute	Femur	Groups treated with higher doses of BMP-2-producing cells were more likely to heal, but also more likely to form heterotopic ossification.	(121)
Johnson et al. (2011)	Rats (*n* = 20)	13 weeks	8 mm	PCL scaffolds and collagen matrix with various combinations of BMP-2 and heparin	Acute	Femur	BMP-2 and heparin in a collagen matrix resulted in new bone formation with mechanical properties similar to intact bone.	(122)
Kaipel et al. (2012)	Rats (*n* = 37)	20–24 weeks	3.8 mm	Fibrin matrix with either PDGF, VEGF, or BMP-2	Chronic (4 weeks)	Femur	The BMP-2 fibrin clot treatment showed a significantly increased union rate and bone volume relative to other groups.	(123)
Kanthan et al. (2011)	Rabbits (*n* = 12)	14–18 weeks	2 cm	Artificial bone grafts (Coragraft) with or without platelet-rich plasma (PRP)	Chronic (3 weeks)	Tibia	The Coragraft with PRP had the best bone healing based on radiological and histological findings.	(124)
Krishnan et al. (2015)	Rats (*n* = 23)	13 weeks	8 cm	Live autograft or nanofiber mesh-alginate (NMA) BMP-2 delivery system	Acute	Femur	The NMA BMP-2 group showed higher total bone volume, bone density, and incidence of bridging compared to the autograft group.	(125)
Li et al. (2011)	Rat (*n* = 24)	12–14 weeks	6 mm	Demineralized bone matrix with either Nel-like molecule-1 (Nell-1) or saline control	Acute	Femur	The Nell-1 treatment groups had increased bone formation compared to the control.	(126)
Minier et al. (2014)	Canine (*n* = 10)	NA	2 cm	Hydrogel/Biphasic calcium phosphate (BCP) with or without BMP-2. Cancellous bone autografts served as positive controls.	Acute	Ulna	The BMP-2-loaded constructs induced mineralized lamellar bone with bridging faster than cancellous-bone autografts.	(127)
Omlor et al. (2016)	Rabbits (*n* = 19)	6 months	15 mm	Gelatin sponge with erythropoietin (EPO) or systemic EPO	Acute	Radius	Both local EPO (gelatin sponge) and systemic EPO showed increased bone formation and callus vascularization.	(128)
Panos et al. (2023)	Rats (*n* = 9)	16 weeks	5 mm	Collagen sponge with human BMP-2	Acute	Femur	BMP-2 treated bones demonstrated inferior mechanical properties compared to those that healed naturally.	(129)
Skaliczki et al. (2013)	Rats (Acute: *n* = 19, Chronic: *n* = 20)	NA	6 mm	Acute and chronic treatment of allografts with and without albumin coat	Acute vs Chronic (4 weeks)	Femur	None of the acute treatments were able to bridge the defect. In the chronic model, the albumin coated group had more bridging and increased trabecular thickness.	(41)
von Rüden et al. (2016)	Human (*n* = 82)	Median = 49 years	NA	Autologous bone grafting with or without BMP-2 and BMP-7	Chronic	Clavicle	96% of the nonunion healed with no dependence on administration of either BMP-2 or BMP-7.	(130)
Wojtowicz et al. (2010)	Rats (*n* = 32)	13–15 weeks	8 mm	PCL scaffolds coated with collagen-mimetic peptide (GFOGER)	Acute	Femur	GFOGER on PCL enhanced bone repair via increased osteoblastic differentiation.	(131)
Yasuda et al. (2012)	Rats (*n* = 60)	7 weeks	8 mm	Frozen stocked allograft coated with BMP-2 paste	Acute	Femur	Allogenic bone grafting with local BMP-2 delivery offered an alternative to autogenous bone grafting.	(132)
Yoon et al. (2014)	Mice (Tibia: *n* = 24, Calvarium: *n* = 18)	6 months	Tibia: 2 mm, Calvarium: 4 mm (diameter)	Collagen sponge with BMP2, Activin A/BMP2 (AB2) chimera, or neither	Acute	Tibia, Calvarium	The AB2 treatment produced more complete healing in both the tibia and calvarium relative to BMP2, even with lower doses.	(133)

### 3.1 Preclinical – Bone Morphogenic Protein

Bone regeneration growth factors, specifically BMP, have been extensively tested using preclinical animal models. Bolander et al. found that BMP-2 with human periosteum-derived cells resulted in tissue formation that mimicked natural fracture healing in mice tibia.^110^ The BMP-2 administration induced the presence of hypertrophic chondrocytes and stimulated mineralized bone bridging, which was confirmed by Hettiaratchi et al. and Johnson et al. in rat femurs.^120,122^ Hettiaratchi et al. found that the use of heparin microparticles with PCL scaffolds improved BMP-2 retention at the nonunion site and spatial localization of bone formation.^120^ Additionally, Johnson et al. found that the new bone formed with administration of BMP-2 and heparin in a collagen matrix had similar mechanical properties to that of intact bone.^122^ It was hypothesized that the addition of heparin caused BMP-2 sequestration at the defect site, decreased diffusion into nearby tissue, and maintained BMP-2 bioactivity to optimize bone regeneration. Conversely, Yoon et al. studied a different additive, activin A (AB2), to promote the bioactivity of BMP-2. They found a synergistic growth effect by using BMP-2 in combination with AB2, whereby superior results were achieved with even lower BMP-2 concentrations relative to BMP-2 alone.^122^ As activin A is a transforming growth factor (TGF), these findings indicate that the optimal usage of growth factors may include a combination of multiple biologically active compounds.^134^

Other studies have found success in a combination treatment of BMP-2 with various grafts. For example, Yasuda et al. examined the ability of BMP-2 to improve bone grafting in rat femurs using frozen bone allografts revealing that the groups with BMP-2 demonstrated improved integration and mechanical strength relative to allografts without BMP-2.^132^ Krishnan et al. used a similar model, but compared the use of BMP-2 with a hybrid nanofiber mesh-alginate delivery system in rat femurs and found the BMP-2 treatment group was superior to standard autograft treatment with respect to bone formation and mechanical function.^125^ Minier et al. also examined effects of BMP-2 in allografts, utilizing a hydrogel composed of biphasic calcium phosphate (BCP) with or without BMP-2 compared to cancellous-bone autografts in canine ulnas.^127^ Their findings support the work by Yasuda et al. and Krishnan et al. as the BMP-loaded constructs induced mineralized lamellar bone with bridging more rapidly than the cancellous-bone autograft controls.^125,127,132^ Yasuda et al. also examined effects of BMP-2 in allografts, utilizing a hydrogel with or without BMP-2, compared to cancellous-bone autografts in canine ulnas.^127^ Their findings supported the work by Yasuda et al. and Krishnan et al. as the BMP-loaded constructs induced mineralized lamellar bone with more rapid bridging compared to the cancellous-bone autograft controls.^125,127,132^ Overall, these studies highlight another treatment modality in combining the BMP-2 growth factor with various graft types.

**Figure 4:**
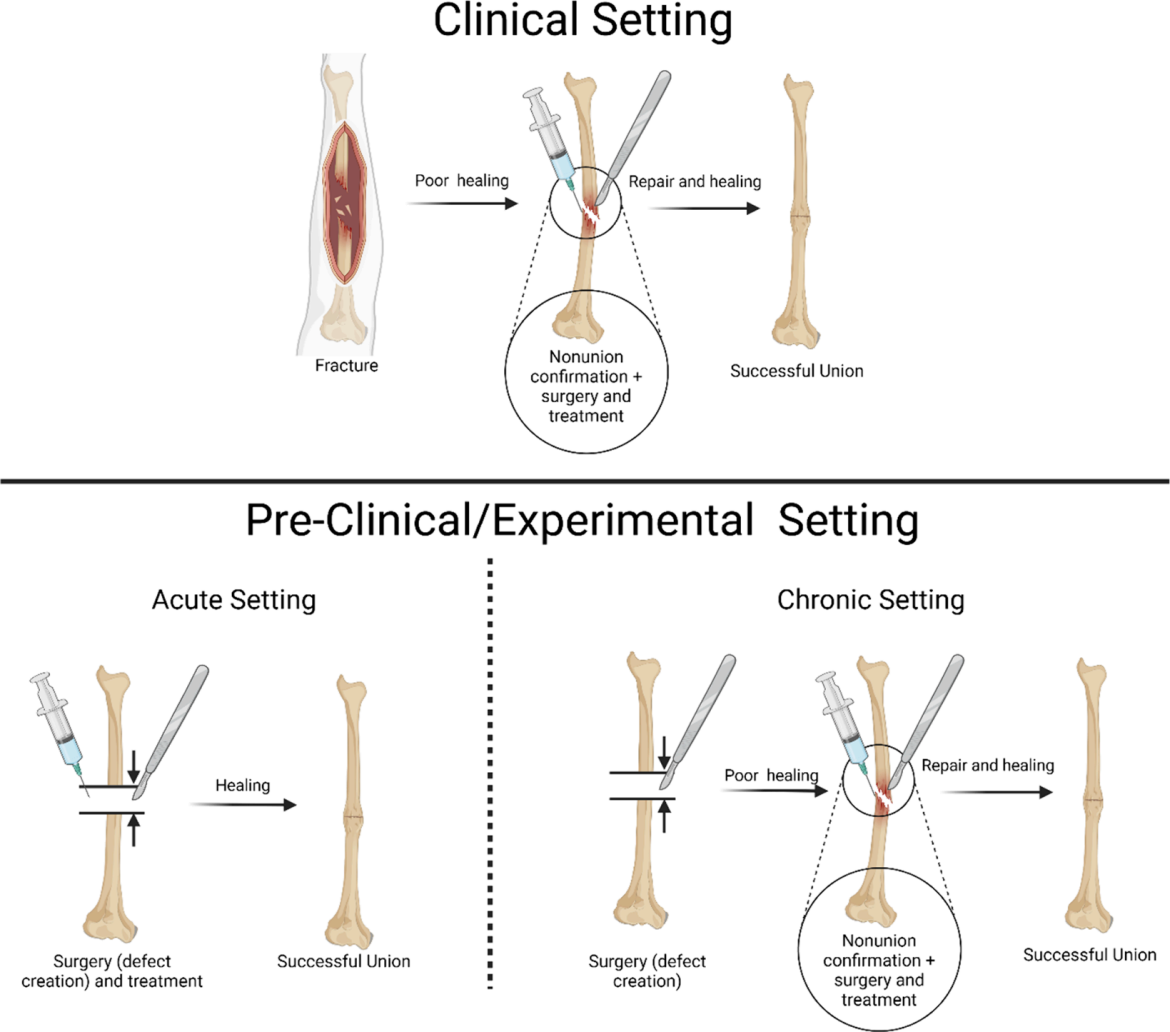
Schematic depicts the experimental design of acute and chronic experimental settings compared to the human (clinical) treatment process. Notably, the chronic experimental design resembles the human treatment course. In the acute case, treatment is administered upon creation of nonunion. However, in the clinical setting, the treatment is administered long after the initial fracture after formation of nonunion. The chronic model better resembles the clinical timeline in which treatment is applied to an already formed nonunion that contains fibrotic tissue and a unique biologic environment. Created in BioRender.com.

The aforementioned studies all utilized a model that consisted of critically sized defect creation followed by immediate treatment. However, this model is inconsistent with the clinical timeline of a chronic nonunion (Figure 4). In order to mimic the clinical treatment of nonunion more accurately, the model should induce a critically sized defect, allocate time for formation of nonunion, and subsequently treat with respective modalities, which to date only a few studies have followed.^102,108,119^ Bosemark et al. investigated the ideal combination of tricalcium phosphate-hydroxyapatite (TCP-HA) scaffold alone, BMP-7 alone, TCP-HA scaffold + BMP-7 combination, or TCP-HA scaffold+ BMP-7+systemic bisphosphonate combination in rat femurs after simulated nonunion for 4 weeks. They identified the combination treatment of TCP-HA scaffold + BMP-7 + systemic bisphosphonate as the best treatment option for healing a critically sized defect as it showed the highest bone volume fraction.^108^ This study was supported by DeBaun et al., who found similar results despite varying treatment delivery. The study utilized a PCL/β-TCP scaffold surrounded by collagen type I sheet, with the addition of microspheres of BMP-2 versus a bare PCL/β-TCP scaffold.^135^ They found that the groups with BMP-2 resulted in complete union, while the bare scaffold did not induce union.^135^ Heckman et al. used a similar model in the treatment of 12–16 week canine radii nonunion. They found that using a poly(DL-lactic acid) and PGA copolymer containing BMP showed significantly increased bone formation relative to implantation without BMP treatment.^119^ Additionally, a study by Kaipel et al. examined the use of fibrin matrix treated with BMP-2 in rat femur nonunion defects. Once again, it was found that the BMP-2 treatment showed a significantly increased union rate and bone volume relative to fibrin matrix alone.^123^ Finally, Franch et al. presented a case of nonunion of a canine radius which was then treated with a 3D-printed β-TCP scaffold and BMP-2 collagen sponge.^136^ They reported that complete healing of the defect was observed 4 months after surgery and the bone plate was able to be removed at 10 months.^136^ Overall, there are a plethora of preclinical studies that point toward the efficacy of BMP as an additive treatment for chronic nonunion.

Though many studies involving the use of BMP-2 for the treatment of nonunion in animals have shown promising results, multiple researchers have reported on limitations of this treatment modality. Ihn et al. found that while BMP-2 did induce femur growth and healing in rats, higher doses were more likely to result in heterotopic ossification.^121^ This potentially serious consequence of BMP-2 raises concerns regarding the balance between inducing bone growth without overstimulation. Furthermore, Panos et al. found that BMP-2 treated rat femurs had inferior mechanical properties indicating that although bone bridging and healing may occur, the resulting tissue may not be equivalent to that of a typical fully healed fracture.^137^ Additionally, several other side effects have been noted from preclinical administration of BMP including, ectopic bone formation, premature suture fusion, rapid local clearance of the biologic, and inappropriate adipogenesis, and bone cysts.^138,139^ These potentially harmful findings pose another hurdle for clinical translation and have slowed the use of BMP in the market.

### 3.2 Preclinical – Emerging Growth Factors

While BMP-2 has shown promise in nonunion treatment, multiple other growth factors (TGF-β, VEGF, erythropoietin (EPO), etc.) have also been studied. TGF-β is a part of the larger family of transforming growth factors of which BMP is also a member.^109,140^ Beck et al. found that bone marrow augmented with TGF-β performed equivalently to autogenous cortical bone grafts in rabbit radii.^109^ Despite this finding, it has also been shown that TGF-β performs inferiorly to BMP as an additive to PLA–PGA implant.^119^ BMP perhaps serves as a more specific activator of bone regeneration compared to the broader proinflammatory effect of TGF-β.

The theory of using proinflammatory molecules to stimulate bone regeneration has also been tested using VEGF. Geiger et al. theorized that regeneration is dependent on the vascularization of bone. In a study of rabbit radii nonunion, it was found that a VEGF gene-activated matrix on a collagen sponge had significantly more bone regeneration with 2–3 times more blood vessel growth compared to controls.^103^ However, the notion that VEGF is the optimal biologic molecule is challenged in a slightly different model. When using a fibrin matrix as a mechanism of delivery, it was found that VEGF was less effective than BMP and failed to adequately stimulate bone healing.^123^

Similar to the theory supporting the use of VEGF, EPO has been explored in the treatment of nonunion due to its osteogenic and angiogenic potency. The possible role of EPO, a common hematopoietic growth factor, in the treatment on nonunion has been examined both systemically and with local application in rabbit radii.^128^ Interestingly, both the locally applied EPO sponge group and the systemic application group demonstrated increases in bone formation and callus vascularization.^128^ However, there are many known side effects of EPO administration, such as but not limited to reports of seizures, hypercoagulability, and hypertension.^128,141^

Platelet Derived Growth Factor (PDGF) is another biologic that has been tested to stimulate bone regeneration in nonunion. DeBaun et al. found that PDGF demonstrated better union in a femoral rat model relative to control, however, complete union was never achieved. Meanwhile, the BMP group was capable of achieving complete union. Kaipel et al. found that a fibrin matrix treated with PDGF did not increase bone healing in a femoral rat model further confirming that PDGF may not be the ideal growth factor in the treatment of nonunion.^123^ However, building on these findings, a recent study by Nayak et al. demonstrated that a mixture of recombinant human PDGF-B chain homodimer combined with bovine type I collagen/β-TCP matrix facilitated complete bridging of the induced chronic nonunion defects in rabbit tibiae, potentiating the use of PDGF in future preclinical trials.^142^

Nerve epidermal growth factor-like protein (Nell-1) is an endogenously secreted protein that binds to many receptors such as protein kinase C, apoptosis-related protein 3, and integrin α3β1. Its osteogenic properties have been extensively studied as a potential therapy for bone regeneration. Historically, it has been applied for craniofacial regeneration and spinal fusion. More recently, Nell-1 has been used to promote the healing of critically sized defects in long bones. Li et al. studied the treatment of femoral rat defects with a demineralized bone matrix implant seeded with Nell-1.^126^ It was concluded that Nell-1 can significantly improve bone regeneration in a dose-dependent manner which proves its efficacy as an osteospecific growth factor.^126,143^

The Wnt pathway regulates cell fate and development, and it has been previously implicated in the bone remodeling pathway. Its ligands are typically secreted glycoproteins, however, exogenous application of Wnt1 has recently been explored as a possible nonunion treatment modality.^118^ Wnt1 is an activator of the Wnt pathway and has been shown to have intense osteoanabolic effects on long bone.^144^ In a femoral mice critically sized defect model, Luntzer et al. applied Wnt1 onto a collagen gel implant, demonstrating increased bone volume and trabecular thickness compared to the control group.^118^ This novel biologic illuminates another approach toward nonunion treatment, yet further studies are required to confirm its efficacy.

While Wnt1 signaling plays a crucial role in bone formation and regeneration, its effects vary depending on the type of ossification. In intramembranous ossification, which occurs in flat bones and during fracture healing of bones under stable mechanical conditions, Wnt/β-catenin signaling promotes osteoblast differentiation and bone formation.^145^ Conversely, in endochondral ossification, which is the primary mode of long bone development and fracture healing under less stable conditions, the role of Wnt1 signaling is more complex. During the early stages of endochondral ossification, Wnt1 signaling must be suppressed to allow for cartilage formation, while in later stages, it promotes the transition from cartilage to bone.^146^ Furthermore, the efficacy of Wnt1 signaling is closely tied to the oxidative state of the cellular environment. High levels of ROS can inhibit Wnt1 signaling progression, potentially impeding bone healing. This interplay between ROS and Wnt1 signaling underscores the importance of managing oxidative stress in the fracture environment to promote optimal healing conditions.^147^ The dual nature of Wnt signaling in different ossification processes and its sensitivity to ROS levels highlight the need for carefully tailored therapeutic approaches when targeting this pathway for nonunion treatment.

Other proteins, while not considered growth factors, have also been studied for their potential to improve bone healing. Skaliczki et al. used a femoral critically sized defect in rats to test the efficacy of allografts coated with albumin for bone regeneration.^41^ They found that the albumin-coated allografts were not able to bridge the defect, however, they did improve the trabecular thickness and pattern factor - an indicator of better-connected trabecular lattices.^41,148^ Additionally, this study utilized an acute and chronic arm to better emulate the clinical treatment of nonunion. It was found that the chronic arm (implantation after 4 weeks of designated healing), created more bridging and increased trabecular thickness in the albumin-coated group relative to the acute arm.^41^ Another study by Wojtowicz et al. also studied critical size defects in rat femurs and found similar results using a PCL scaffold with collagen-mimetic peptide as an osteogenic protein.^131^ It is theorized that collagen-mimetic peptide can aid in bone regeneration due to its role in osteoblastic differentiation.^149^ The results presented by Wojtowicz et al. showed faster and increased bone formation, yet not significantly better bone quality.^131^ Therefore, while these proteins exhibit promise in accelerating bone formation, their lack of bridging and mechanical stability demonstrates inferiority to other growth factors for the treatment of chronic nonunion.

Finally, another biologic molecule with promising preclinical efficacy is dipyridamole (DIPY), an indirect adenosine agonist.^150^ Its mechanism is well-described in literature: it inhibits phosphodiesterase, adenosine deaminase, and equilibrative nucleoside transporter 1 (ENT-1), resulting in increased extracellular concentrations of adenosine.^72,150^ Adenosine is known to increase osteoblast differentiation, inhibit inflammation, and increase intracellular concentrations of cyclic adenosine monophosphate and cyclic guanosine monophosphate.^72,151^ With this extensive mechanism, DIPY is theorized to aid in bone regeneration. Witek et al. studied the effect of DIPY in bone regeneration using a rabbit radii critically sized defect model.^152^ A bioactive ceramic implant composed of β-TCP was immersed in various concentrations (10 μM, 100 μM, or 1000 μM) of DIPY and inserted into the defects. The DIPY-immersed implants outperformed control groups, leading into an increase in bone volume in a dose-dependent manner while maintaining the structural integrity of native bone.^152^ These results have been reproduced in other anatomic locations, showing promise for utility in both craniofacial and orthopedic applications.^70,153^ Overall, DIPY has preclinically shown to be effective in the treatment of lone bone critically sized defects and serves as another option in the multitude of biologics that may aid in bone regeneration for the treatment of chronic nonunion, and it remains to be evaluated.

### 3.3 Clinical – BMP

In addition to its use in preclinical models, BMP has also been studied in the clinical setting. Donegan et al. explored the use of BMP-2 in the masquelet technique.^112^ This two-step technique first places a spacer in a long bone defect to induce a foreign body reaction followed by a second operation 6 to 8 weeks later, filling the defect with bone graft.^154^ In this study, 11 human patients were treated with a combination of BMP-2 and platelet rich concentrate. Osseous consolidation and complete healing were observed in 10 of the 11 patients.^112^ These results are supported by Crawford et al., who examined the use of a locking plate with BMP administration.^111^ Out of nine patients being treated for humeral diaphysis atrophic nonunion, eight demonstrated signs of union at one year.^111^ The results of these studies are consistent and confirm the efficacy of BMP as a treatment modality for nonunion without the use of a bone graft.

Other studies have analyzed the effect of BMP either in conjunction with, or versus, a bone graft. Giannoudis et al. studied autologous bone grafts with or without BMP-7 for the treatment of various chronic humerus, femur, and tibia nonunion.^117^ It was found that the combination group had a high level of success with 100% of patients achieving union, suggesting that the combination treatment may be more effective and economical than autologous bone graft alone.^117^ While Giannoudis et al. reported a possible synergistic effect of BMP-7 with autologous bone grafting, von Ruden et al. reported contradicting results.^117,155^ They found, in their investigation of the treatment of clavicle nonunion, that successful union was not dependent on the use of BMP-2 or BMP-7.^117,155^ On the other hand, Friedlaender et al. compared the use of internal fixation with BMP-7 and collagen type I versus internal fixation with autograft in chronic nonunion of the human tibia.^156^ Friedlaender et al. demonstrated that the BMP-7 and collagen type I treatment had comparable rates of success to the autograft, although without added donor site morbidity associated with harvesting the autograft.^156^ However, there is also contradicting evidence that BMP is not adequate for clinical treatment. Ekrol et al. studied the use of BMP-7 versus autogenous bone graft harvested from the ipsilateral iliac crest in distal radius nonunion, where BMP-7 was found to be an inferior treatment with slower healing rate and decreased stability postoperatively.^113^ Overall, the literature supporting the use of BMP with or without bone grafting is inconsistent and further clinical research is necessary to determine the optimal treatment regimen.

Despite these strides in the clinical application of growth factors for bone regeneration, there are many cited limitations and side effects of BMP in humans. For example, the use of BMP can cause an excessive inflammatory response that has been reported to result in seroma formation and cervical spine swelling, leading to radiculopathy.^157,158^ There have also been reports of both ectopic bone formation and osteoclast activation leading to bone resorption.^158,159^ Finally, the administration of BMP has potential to impair wound healing, leading to hematoma, wound dehiscence, or infection.^139,160^ While BMP and other growth factors have shown promising efficacy in the treatment of chronic nonunion, these complications must be considered before their clinical administration.

### 3.4 Exogenous Antioxidants

While not technically growth factors, exogenous antioxidants are bioactive compounds which have demonstrated the ability to promote bone regeneration by combating the detrimental effects of ROS, thereby potentially aiding in nonunion treatment. Vitamins, particularly vitamin C (ascorbic acid) and vitamin E (tocopherol) play crucial roles in bone formation. Vitamin C is essential for collagen synthesis and acts as a cofactor for enzymes involved in bone matrix formation. In a previously conducted review, effects of vitamin C showed a positive effect on trabecular bone formation by influencing expression of bone matrix genes in osteoblasts.^161^ Vitamin E, a potent antioxidant, has been shown to protect against oxidative damage in bone cells. Nazrun et al. found that vitamin E supplementation improved fracture healing in osteoporotic rats, enhancing callus volume and strength.^162^ N-acetylcysteine (NAC), a precursor to the potent antioxidant glutathione, has demonstrated the ability to enhance osteoblast differentiation and mineralization while reducing oxidative stress. Yamada et al. found that NAC treatment improved bone healing in a rat femoral fracture model by promoting osteoblast differentiation and reducing inflammation.^163^ Imaging demonstrated that the addition of NAC to a collagenous sponge implanted into the critically sized femoral defect yielded acceleration and completion of defect closure after a 6-week time period.^163^ Resveratrol, a polyphenol found in grapes and berries, has shown promise in stimulating osteoblast activity and inhibiting osteoclast formation, potentially accelerating bone healing. In an animal study, Casarin et al. found that resveratrol enhanced the repair of critically sized bone defects as well as the mechanical retention of titanium implants in a rodent model.^164^

While these compounds show potential as adjunct therapies in nonunion treatment, further clinical research is needed to establish optimal dosing, timing, and delivery methods in the context of fracture healing. The complex interplay between antioxidants, oxidative stress, and bone metabolism underscores the need for carefully designed studies to fully elucidate their role in nonunion treatment.

## 4 Stem Cells

Addressing bone nonunion, a noteworthy avenue of exploration, centers on stem cells and their derivatives. In the realm of stem cell research, particular focus has been directed toward mesenchymal stem cells (MSCs). These stem cells are stromal cells endowed with the capacity for self-renewal and multilineage differentiation.^165^ These cells can be extracted from various tissues, such as the umbilical cord, bone marrow, adipose tissue, among others, and have been widely used in different tissue regeneration studies.^165^ In the context of these MSCs, comprehensive insights and findings can be derived mainly from preclinical studies. Table 3 presents the details of the analyzed preclinical studies.

**Table 3:** Stem Cells and Derivatives for the Treatment of Nonunion in Long Bones

Author	Subjects	Age	Size of Defect	Product	Administration Time	Site of Nonunion	Results	References
Bernhard et al. (2017)	Rats (*n* = 28)	NA	5 mm	Human adipose stem cells differentiated into hypertrophic chondrocytes in decellularized bone scaffolds.	Acute	Femur	Rats without any implant had nonunion and those with the hypertrophic chondrocyte stem cell scaffold had enhanced regeneration relative to osteoblast stem cell scaffold.	(166)
Decambron et al. (2017)	Sheep (*n* = 18)	2 years	25 mm	Cube-shaped coral granules loaded with either BMP-2, autologous bone-marrow derived mesenchymal stem cells (MSC), both, or without loading.	Acute	Metatarsal	Scaffolds with dual delivery of BMP-2 and autologous bone-marrow-derived mesenchymal stem cell had increased bone formation, though no significant improvement.	(167)
Dilogo et al. (2017)	Human (*n* = 1)	54 years	12 cm	Umbilical cord-derived mesenchymal stem cells (UC-MSCs) with BMP-2, hydroxyapatite granules, and mechanical stabilization.	Chronic (14 months after initial fracture)	Femur	Infected, chronic nonunion fractures may be treated with a combination of UC-MSC, hydroxyapatite, and BMP-2 to deliver growth and osteoinductive factors.	(168)
Guo et al. (2006)	Rabbits (*n* = 18)	NA	15 mm	Basic fibroblast growth factor (bFGF) gene modified MSCs on biodegradable porous β tricalcium phosphate (β-TCP) ceramics.	Acute	Radius	bFGF gene modified MSCs increased osteogenic properties and stimulated vascular regeneration to supply new bone formation.	(169)
Maiti et al. (2018)	Rabbits (*n* = 36)	7 months	15 mm	Autogenous, allogenic, ovine, and canine bone marrow-derived mesenchymal stem cells (BMSCs) were seeded onto tricalcium phosphate.	Acute	Radius	Autogenous, allogenous, and xenogenous BMSC-seeded bioscaffolds showed significantly faster tissue formation compared to nonseeded control scaffold.	(170)
Meinel et al. (2006)	Rats (*n* = 24)	NA	5 mm	Silk scaffolds with either osteoblastic human mesenchymal stem cells (hMSCs), undifferentiated hMSCs, or no seeding.	Acute	Femur	The silk scaffolds with osteoblastic hMSCs produced greater bone volumes, greater maximal load and torque, and demonstrated osteogenic ability relative to other treatment groups.	(171)
Ninu et al. (2017)	Rabbits (*n* = 18)	6 months -1 year	30 mm	Silica coated calcium hydroxyapatite bioceramic with or without seeding via BMSCs.	Acute	Radius	BMSCs promoted faster healing of critically sized defects relative to baseline.	(172)
Peters et al. (2009)	Rats (*n* = 160	NA	10 mm	Percutaneous injection of mesenchymal stem cells (MSCs) or bone marrow-derived osteogenic predifferentiated cells (OCPs).	Acute	Femur	Locally injected OCPs enhanced healing in a critically sized bone defect and provided a minimally invasive approach to healing nonunion.	(173)
Rai et al. (2010)	Rats (*n* = 6)	4–6 weeks	8 mm	Human MSC seeded into polycaprolactone-20% tricalcium phosphate (PCL-TCP) scaffolds.	Acute	Femur	The efficacy of using PCL-TCP seeded with human MSCs was highly variable and did not produce a consistent healing response.	(174)
Romero et al. (2017)	Mice (*n* = 52)	6–8 week	4 mm	Allografts with polysaccharide-based tissue-engineered periosteum with FGF-2, TBF-β1, and adipose-derived MSCs.	Acute	Femur	The treatment provided a nonsignificant increase in normalized bone callus volume with inhibition of allograft incorporation, alluding to the need for further treatment optimization.	(175)
Schubert et al. (2013)	Pigs (*n* = 6)	6 months	Lumbar fusion | 4 mm	PEEK fusion cage with 3D osteoblastic differentiated adipose-derived mesenchymal stem cells (AMSCs) | Osteoblastic AMSC autograft.	Chronic (3 months | 6 months)	Spine | Femur	The 3D osteoblastic adipose MSC graft demonstrate potential to form new bone. The 3D engineered osteoplastic AMSC autograft can induce bone fusion in nonunion.	(176)
Tam et al. (2021)	Mice (*n* = 4)	NA	4 mm	Orthotopic implant of chondrogenic-differentiated human pluripotent stem cells (PSCs).	Acute	Tibia	Orthotopically implanted PSCs can recruit osteogenic precursors for bone repair and induce bone bridging.	(177)
van der Stok et al. (2014)	Rats (*n* = 27)	16 weeks	6 mm	Graft of chondrogenically differentiated and undifferentiated human bone marrow-derived MSCs.	Chronic (6 weeks)	Femur	Chondrogenically differentiated MSC grafts had significantly more bone regeneration relative to undifferentiated MSC grafts.	(178)
Zamani Mazdeh et al. (2018)	Rabbits (*n* = 20)	NA	Anatomically variable	Fibrin clot with bone marrow-derived mesenchymal stem cells (BMSCs) and 17β-estradiol.	Acute	Radius	*In situ* 17β-estradiol with BMSCs improved osteogenic capacity and accelerated bone healing relative to other groups.	(179)

### 4.1 Acute Administration

Preclinical studies performed in different animal models exploring the application of stem cells as a treatment for bone nonunion, have predominantly focused on acute time administration. However, there are also studies available that assess the efficacy of chronic administration.

Regarding the acute administration of stem cells, Guo et al.,^169^ conducted a study utilizing basic fibroblast growth factor (bFGF) gene-modified MSCs on biodegradable porous β-TCP ceramics within the rabbit radii. Their findings underscored the augmentation of osteogenic properties, and the stimulation of vascular regeneration facilitated by bFGF-modified MSCs. Additionally, Romero et al., investigated a combined therapeutic approach involving allografts and a polysaccharide-based tissue-engineered periosteum. This engineered periosteum delivered Fibroblast Growth Factor-2 (FGF-2), TBF-β1, and adipose-derived MSCs into the femurs of mice. Despite observing a nonsignificant improvement in bone callus, the treatment group exhibited a noteworthy hindrance in the incorporation of the allograft.^180^ These studies collectively advance our comprehension of acute stem cell interventions, emphasizing the significance of tailored modifications and highlighting the intricate interplay between various components in regenerative strategies.

On the other hand, pertaining to acute administration of bone-marrow-derived mesenchymal stem cells (BMSCs), Maiti et al. evaluated various types of MSCs, including autogenous, allogeneic, ovine, and canine bone marrow-derived MSCs, in the seeding of HA/TCP scaffolds for the treatment of rabbit radii defects.^170^ The bioscaffolds, seeded with autogenous, allogeneic, and xenogeneic MSCs, exhibited a markedly accelerated formation compared to the control scaffold that was not seeded. These results align with the findings of Ninu et al.,^172^ who conducted an investigation into the seeding of silica-coated calcium hydroxyapatite bioceramic with BMSCs. Their study revealed that the seeding of BMSCs facilitated a swifter healing process for critically sized defects in rabbit radii. In addition, Zamani Mazdeh et al.^181^ conducted an examination on rabbit radii, where they evaluated the application of BMSCs in conjunction with 17β-estradiol, which has been shown to improve the osteogenesis and proliferation potential of the MSCs via estrogen receptors. Their findings indicate that the *in situ* application of 17β-estradiol with BMSCs not only enhanced osteogenic capacity but also expedited the process of bone healing.

### 4.2 Chronic Administration

Regarding the studies that carried out chronic administration, Peters et al.,^173^ explored the efficacy of a percutaneous injection involving MSCs or bone marrow-derived osteogenic predifferentiated cells (OCPs) in rat femurs. This experimental setup involved cauterization of the periosteum and removal of bone marrow to simulate a nonunion scenario. The findings revealed that the OCPs cohort exhibited improved healing across all outcome measures compared to the control group, whereas the MSC cohort did not demonstrate similar enhancements. Building on this, Van der Stok et al., delved into the comparison between undifferentiated MSC (un-MSC) pellets and chondrogenically differentiated MSC (ch-MSC) pellets.^178^ Their study aimed at inducing bone regeneration in critically sized defects in rat femurs that had been left untreated for 6 weeks to simulate nonunion. The results showed a significant disparity, with the ch-MSC pellets outperforming the un-MSC pellets in terms of bone regeneration. This underscores the importance of considering the differentiation status of MSCs in such regenerative contexts.

Furthering our understanding of this topic, Schubert et al. explored the potential of osteoblastic differentiated adipose mesenchymal stem cells (AMSCs).^176^ Their study focused on pigs with artificially induced nonunion, confirmed through CT scans after 6 months. Remarkably, the AMSC model led to bone fusion within a previously fibrotic environment characterized by poor vascularization. In summary, these studies collectively contribute valuable insights into the nuanced dynamics of chronic administration of regenerative cells, emphasizing the role of differentiation status and providing crucial benchmarks for evaluating healing outcomes in simulated nonunion scenarios.

While MSCs show great promise in nonunion treatment, several clinical concerns must be addressed. Although MSCs are generally considered to have low immunogenicity, immune rejection remains a potential risk, especially with allogeneic transplants.^182^ Furthermore, long-term effects of MSC therapy are not fully understood, and there are concerns about their persistence and potential for uncontrolled differentiation. Safety is a paramount concern, with the theoretical risk of tumor formation being the most serious. While not directly causing tumor formation on their own, MSCs can significantly contribute to tumor development and progression.^183^ Additionally, the optimal source, dosage, and delivery method of MSCs for nonunion treatment remain to be standardized. Future research must focus on addressing these clinical concerns through long-term follow-up studies, improved cell tracking methods, and standardization of protocols to ensure the safe and effective use of MSCs in nonunion treatment.

## 5 Conclusion and Limitations

The use of growth factors and stem cells, as well as novel mechanisms of delivery, continue to demonstrate promising results in the treatment of long bone nonunion. Key takeaways from each section contribute significantly to the broader goal of improving bone regeneration therapies for nonunion. BMP-2 and VEGF play crucial roles in stimulating osteogenesis and angiogenesis, respectively, addressing critical aspects of bone healing. MSCs have shown promise in enhancing bone regeneration through their multilineage differentiation potential and paracrine effects, although standardized protocols and long-term safety studies are essential. Various biomaterials, including natural polymers, bioceramics, and synthetic polymers, have been evaluated as delivery vehicles for growth factors and stem cells, each offering unique advantages such as biocompatibility, mechanical strength, or customizability. Additionally, it remains uncertain whether specific treatment modalities are more effective at certain anatomic locations based on the local healing environment. It is also unclear whether specific risk factors (smoking, vitamin D deficiency, etc.) alter the efficacy of aforementioned treatments. Furthermore, due to a large number of studies utilizing an acute defect model which likely varies from a true nonunion (biologically), there is a need for more research into these treatment modalities in a chronic nonunion model to accurately emulate the clinical timeline. It is also important to note the limitations of animal models in this study and the challenges of translating these findings into human applications. Animal models may not fully replicate the complex biological and mechanical environment of human nonunion, and factors such as differences in bone metabolism and healing rates between species must be considered. Additionally, the controlled conditions of preclinical studies may not account for the variability in patient factors and comorbidities encountered in clinical practice. Based on current preclinical results, these modalities may be able to shorten nonunion duration, accelerate the healing process, and better resemble native bone. Nevertheless, there is a need for further randomized clinical trials in humans using these modalities to confirm preliminary findings and treatments in a patient population. Optimistically, these treatments could be used in complex fractures with nonunion risk factors to stimulate bone regeneration and prevent the formation of a nonunion. In doing so, this would relieve economic burden, mitigate the various complications that arise with nonunion, and alleviate patient suffering with quicker recovery.
